# Genetic Analysis and Fingerprint Construction for Thick-Skinned Melon (*Cucumis melo* subsp. *melo*) Based on InDel Markers

**DOI:** 10.3390/plants14243782

**Published:** 2025-12-12

**Authors:** Dandan Ren, Jinglei Liao, Keyan Zhang, Jiaying Zhang, Jingtao Qu, Guobin Ma, Jufen Li

**Affiliations:** 1 Shanghai Key Laboratory of Protected Horticultural Technology, Horticultural Research Institute, Shanghai Academy of Agricultural Sciences, Shanghai 201403, China; rendandan@saas.sh.cn (D.R.); 20210401@saas.sh.cn (K.Z.); jiayingzsaas@163.com (J.Z.); qujingtao@saas.sh.cn (J.Q.); maguobin2006@163.com (G.M.); 2 School of Life Sciences, East China Normal University, Shanghai 200241, China; 51261300012@stu.ecnu.edu.cn

**Keywords:** *Cucumis melo* L., InDel markers, population structure, DNA fingerprinting, cultivar discrimination

## Abstract

Melon (*Cucumis melo* L.) is a significant horticultural crop valued for its aroma and health-promoting compounds. However, the genetic similarity among numerous varieties poses challenges for identification and breeding. ‘Dongfangmi No.4’ is an F_1_ hybrid derived from a cross between two Hami melon inbred lines, ‘M06-1-3’ and ‘M15-3’. This study utilized resequencing data derived from the bi-parents of ‘Dongfangmi No.4’ to identify 557,878 insertion and deletion (InDel) variations across the entire genome. Thirty-nine highly polymorphic InDel markers were screened to conduct a genetic analysis of 40 representative cultivated varieties, with marker MS108 specifically distinguishing ‘Dongfangmi No.4’ from the other 39 cultivated varieties. Genetic analysis revealed a high level of genetic diversity within the population (average observed heterozygosity Ho = 0.313, Shannon index I = 0.528), and polymorphic information content (PIC) analysis indicated that 54% of the markers (21/39) were highly polymorphic. Principal component analysis (PCA) and clustering demonstrated significant genetic differentiation between cantaloupe and Hami melons, as well as between cantaloupe and honeydew. In contrast, the genetic boundaries between Hami melons and honeydew were obscured due to frequent germplasm exchange. Ultimately, seven core InDel markers were selected to construct the DNA fingerprinting map, successfully achieving complete differentiation of 40 varieties. This marker system provides an effective molecular tool for melon variety identification, intellectual property protection, and breeding.

## 1. Introduction

The melon (*Cucumis melo* L.) is a significant horticultural crop within the Cucurbitaceae family, valued by consumers for its rich nutritional content and sweet flavor. This species is widely distributed across tropical and subtropical regions, with its origins believed to lie in Africa and Asia [1,2]. Through extensive global cultivation, it has undergone considerable natural variation, particularly in fruit characteristics such as size, shape, skin color, sugar content, acidity, texture, and aroma, exhibiting a wide range of phenotypic diversity [3,4]. Currently, China is the largest producer and consumer of melons worldwide. According to FAO data (http://www.fao.org/faostat, accessed on 1 March 2025), global melon production in 2023 reached 28.55 million tons, with China contributing 14.25 million tons, approximately half of the global total. Additionally, China represents the largest global melon seed market, with a substantial number of variety registrations, presenting significant challenges for the accurate evaluation, differentiation, and breeding innovation of varieties [5,6]. In particular, ‘Dongfangmi No. 4’, a widely cultivated F_1_ hybrid melon valued for its high quality and productivity, faces serious issues of variety counterfeiting in the market, leading to economic losses and hindering breeding innovation. Effective molecular tools are urgently needed for its identification and protection.

Melons are generally classified into two subspecies, *C. melo* subsp. *melo* and *C. melo* subsp. *agrestis* [7], and can be further categorized into thin-skinned and thick-skinned varietal groups based on pericarp characteristics. Market-significant cultivated varieties such as Hami melon, honeydew, and cantaloupe all belong to the *C. melo* subspecies (thick-skinned group), commonly referred to as muskmelon. While these varieties can be easily distinguished based on morphological differences observed at the mature fruit stage, accurate identification during the seedling, plant, or seed stages presents significant challenges [8]. Therefore, the development of genotypic markers that can rapidly and accurately differentiate various melon types and their varieties at the DNA level is essential for cultivar identification, seed purity testing, and the breeding process.

The DNA fingerprinting technique based on molecular markers has been established as a rapid, accurate, and effective method that is not influenced by growth environment or developmental stage [9]. DNA fingerprints were constructed from resequencing data of 149 melon varieties, ultimately selecting 23 Simple Sequence Repeat (SSR) and 40 Single Nucleotide Polymorphism (SNP) markers as the core marker set, which effectively distinguished between melon subspecies [8]. Sadeghpour et al. [10] conducted a study that combined SSR, Inter-Simple Sequence Repeat (ISSR), and Sequence-Related Amplified Polymorphism (SRAP) markers to analyze the genetic structure of *Fusarium* wilt-resistant varieties, demonstrating the advantages of SSR markers in terms of polymorphism information content (PIC) and discrimination efficiency. In another study, Flores-León et al. [11] analyzed 47 Spanish melon germplasms, identifying a total of 66,971 high-quality SNPs. Despite the extensive use of SSR and SNP markers for melon variety identification and genetic diversity analysis, studies employing Insertion and Deletion (InDel) markers remain relatively limited.

InDel markers refer to DNA length polymorphism sites formed by small fragments (usually 1–50 bp) of insertions or deletions at specific positions among different individuals in the genome. In plants, InDels represent a substantial source of genetic variation, influencing gene expression and phenotypic traits, and account for approximately 10–15% of total genomic variation [12]. These markers are widely distributed throughout the genome and are typically caused by specific cellular mechanisms, including the movement of transposable elements, replication slippage, and unequal crossing over [13]. InDels are easily detectable and can be analyzed using conventional PCR in combination with agarose or polyacrylamide gel electrophoresis [14,15]. Furthermore, InDel markers have been extensively applied in various fields of plant and animal research, including fingerprinting, genetic diversity assessment, population structure analysis, core germplasm screening, linkage map construction, QTL (Quantitative Trait Locus) mapping, and molecular-assisted breeding. In the study of melon powdery mildew resistance, the candidate region was narrowed down to 63.5 kb using InDel markers. Among these markers, chr06_indel_5,047,127 effectively identified resistant materials in both the F_2_ population and 30 inbred lines [16]. In cucumber sex identification, the sex-linked InDel marker located at Chr 2: 102,799,917–102,799,933 bp successfully distinguished plant sex [17].

This study focuses on 40 representative thick-skinned melon varieties commonly found in the Chinese market. Utilizing filtered InDel markers derived from the resequencing data of ‘Dongfangmi No.4’ and its bi-parental lines, we performed cluster analysis and constructed DNA fingerprints for these tested samples. The research aims to thoroughly investigate the genetic relationships among these varieties and establish a practical and efficient identification system. The findings are expected to provide significant molecular evidence for distinctness, uniformity, and stability (DUS) testing, as well as for the protection of variety rights of thick-skinned melons, demonstrating substantial practical application value.

## 2. Results

### 2.1. The Chromosomal Distribution Characteristics of InDel Variations

**Figure 1:**
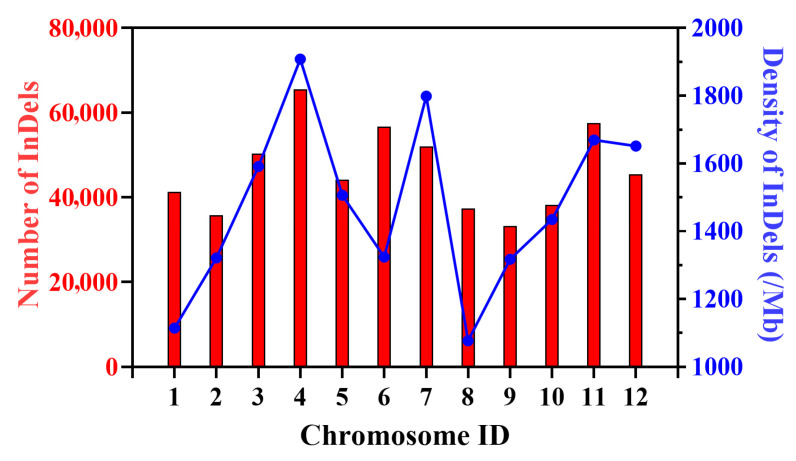
Number of InDels, and Genome-wide Density of InDels (/Mb) for each of the 12 chromosomes.

Based on the resequencing results of the bi-parents of ‘Dongfangmi No.4’, a total of 557,878 InDel variants were identified. Analysis of the InDel length distribution revealed that these variants ranged from 1 bp to 64 bp in length, with the majority being short InDels (1–6 bp). This category accounted for over 85% of the total, with single-nucleotide InDels being the most abundant. Longer InDels (≥10 bp) represented a smaller proportion but are particularly valuable for the development of gel-based markers due to their easier detection (Table S1). These InDels were distributed across all 12 chromosomes, with the number per chromosome ranging from 33,258 on chromosome 9 to 65,498 on chromosome 4. The average InDel density between the bi-parents was calculated to be 1486.25 InDels/Mb. Notably, chromosomes 4 and 7 exhibited higher InDel densities, measuring 1908.47 and 1798.93 InDels/Mb, respectively, while chromosomes 1 and 8 displayed lower densities of 1114.38 and 1077.10 InDels/Mb (Figure 1 and Table S2).

### 2.2. Development and Screening of InDel Markers

**Figure 2:**
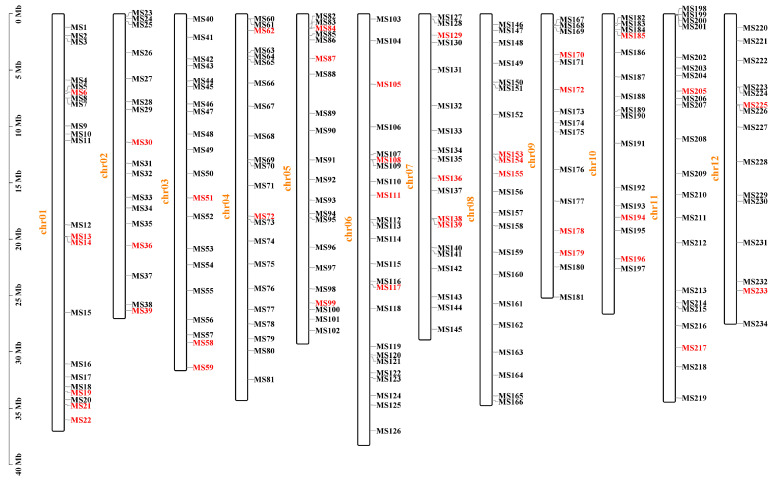
The distribution of 234 InDels on the 12 melon chromosomes. The 39 primer pairs highlighted in red represent the final set of polymorphic markers selected for genetic analysis.

**Figure 3:**
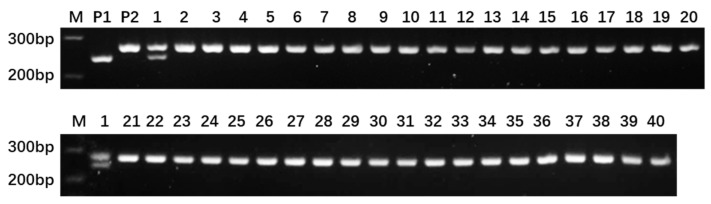
Amplification bands of MS108 primer. Lanes 1–40 correspond to the 40 thick-skinned melon varieties listed in Section 4.1, with lane 1 representing the variety ‘Dongfangmi No.4’. M: 500 bp DNA ladder (key bands at 200, 300, and 400 bp are labeled).

Based on the resequencing data of the parental lines of ‘Dongfangmi No.4’, a selection of 234 random InDel primer pairs designed from these variants, resulted in the identification of 39 pairs exhibiting clear amplification bands and polymorphism when screened across a panel that included ‘Dongfangmi No.4’ and other melon varieties, the substantial reduction from sequenced InDel variants to the final set of validated markers is attributed to stringent selection (Figure 2 and Table 1). Further investigation revealed that at the MS108 locus (chr06_12951796), ‘Dongfangmi No.4’ (P1) exhibits an insertion-type variation, while the other 39 tested cultivars and the parental line P2 are in a homozygous, consistent non-variant state (Figure 3; detailed amplification profiles are provided in Table S3). Consequently, marker MS108 can effectively distinguish ‘Dongfangmi No.4’ and its progeny from these 39 varieties. It is important to note that this marker’s diagnostic utility is specific to this comparative set.

The markers are listed by their assigned laboratory codes (MS number) for consistent reference in this study.

### 2.3. Genetic Diversity Analysis

A genetic diversity analysis was conducted on 40 melon varieties utilizing 39 polymorphic InDel markers identified through screening. A total of 78 alleles were detected, resulting in an average number of alleles (Na) of 2 and an average effective number of alleles (Ne) of 1.608. The overall average observed heterozygosity (Ho) was 0.313. Shannon’s information index (I) ranged from 0.067 to 0.693, with an average of 0.528, indicating that the tested materials exhibit a high level of genetic diversity (Table 2).

**Figure 4:**
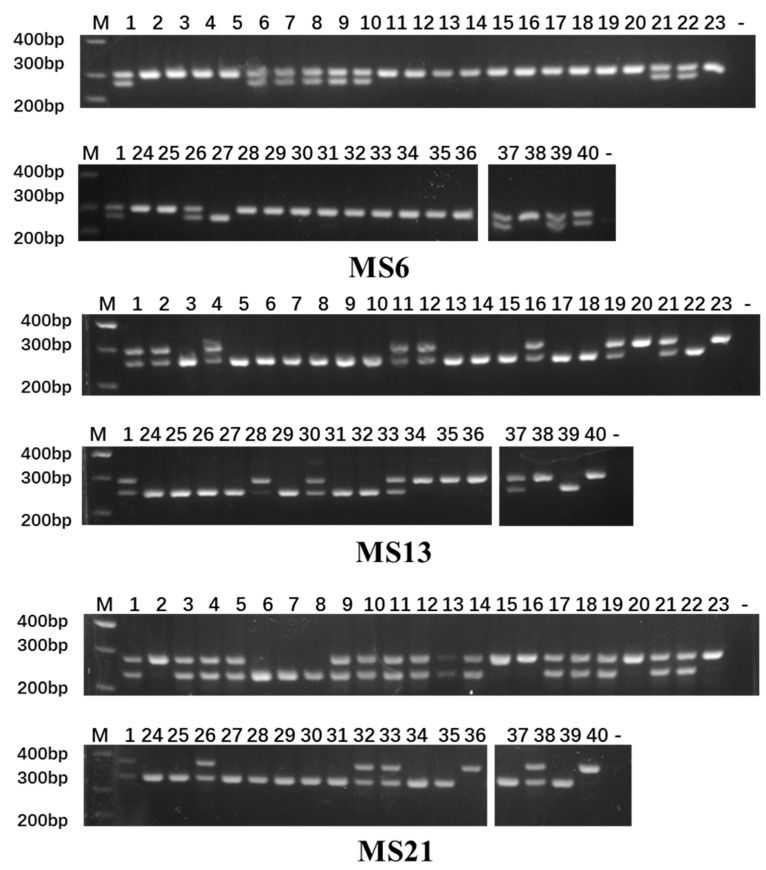
Validation of a subset of polymorphic InDel markers. Gel images show amplification results for representative markers from different chromosomes. Lanes 1–40 correspond to the varieties listed in Section 4.1. M: 500 bp DNA ladder (key bands at 200, 300, and 400 bp are labeled).

The polymorphism level of the loci was assessed based on the Polymorphism Information Content (PIC): PIC > 0.5 indicates high polymorphism, 0.25 ≤ PIC ≤ 0.5 indicates moderate polymorphism, and PIC < 0.25 indicates low polymorphism. Among the 39 InDel markers, 21 loci displayed high polymorphism, while 15 exhibited moderate polymorphism. Notably, MS62 had the highest PIC value (0.664), indicating the greatest genetic variation, whereas MS108 had the lowest PIC value (0.048) (Table 2). The average minor allele frequency (MAF) across all InDel markers was 0.267, with values ranging from 0.010 to 0.488, indicating a high degree of polymorphism among these markers. The amplification results for the selected primers are illustrated in Figure 4.

### 2.4. Genetic Differentiation

**Figure 5:**
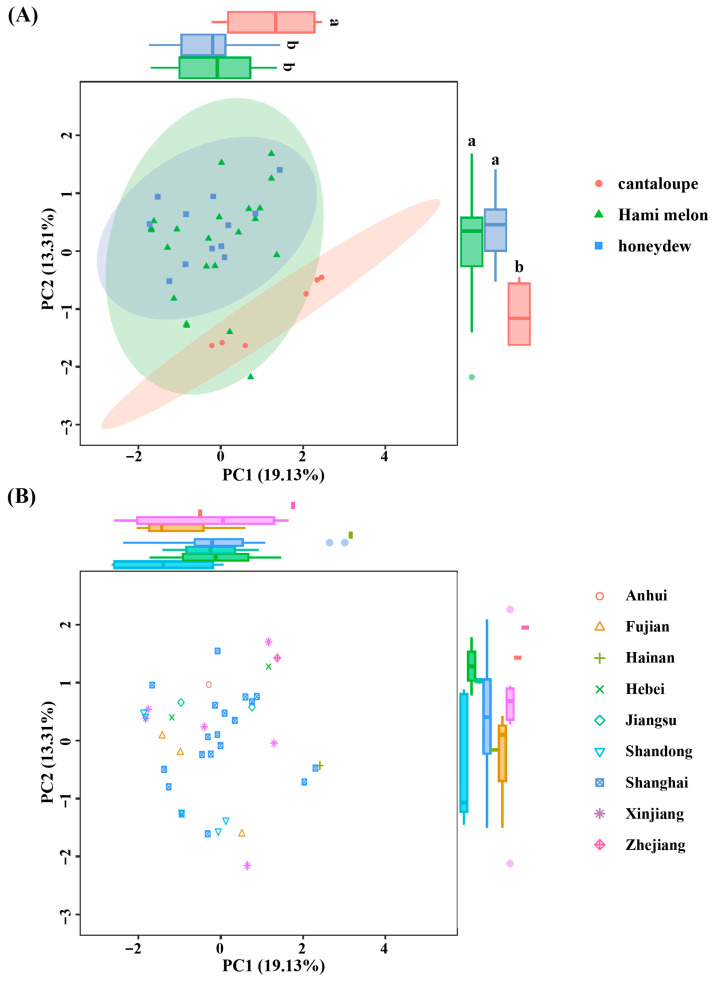
The principal analysis (PCA) score plot depicting the distribution of 40 melon varieties. Individuals are color-coded with melon types (**A**) and geographical origin (**B**). Significant differences (*p* < 0.05) are indicated by different letters.

Based on the allele data, Principal Component Analysis (PCA) revealed that the first two principal components (PC1 and PC2) collectively accounted for 32.44% of the genetic variation, with PC1 contributing 19.13% and PC2 contributing 13.31%. The genetic differences between the Hami melon and honeydew types were relatively minor; however, significant clustering separation was observed in the cantaloupe type (Figure 5A). Furthermore, no clear association was identified between the genetic relationships revealed by PCA and the geographical origins of the accessions (Figure 5B). This lack of phylogeographic structure is not uncommon in extensively cultivated and commercially exchanged crops. It likely reflects the combined effects of modern breeding practices, which prioritize specific traits over geographic provenance, leading to widespread germplasm exchange and genetic homogenization across regions.

**Figure 6:**
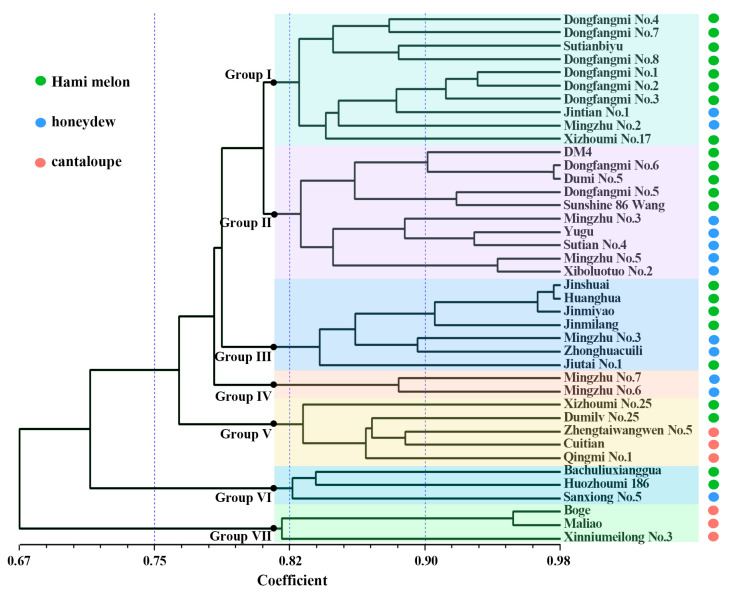
UPGMA dendrogram of 40 melon varieties based on genetic distances calculated from 39 InDel markers. Varieties are colored according to their type: Hami melon (green), honeydew (blue), and cantaloupe (pink). The seven major clusters (Group I–VII) were defined at the threshold of coefficient > 0.80, as indicated by the vertical dashed line. Branch support was assessed using 1000 bootstrap replicates, and values above 50% are shown at the corresponding nodes.

The clustering analysis based on the Unweighted Pair Group Method with Arithmetic Mean (UPGMA) method divided the 40 materials into 7 subgroups (Figure 6):

Group I: includes 8 Hami melon types and 2 honeydew types;

Group II: Includes 5 Hami melon types and 5 honeydew types;

Group III: includes 5 Hami melon types and 2 honeydew types;

Group IV: Includes two varieties of honeydew (Mingzhu No. 6, Mingzhu No. 7), both of which feature golden-yellow skin;

Group V: Includes 3 cantaloupe types (Zhengtaiwangwen No. 5, Cuitian, Qingmi No. 1) and two Hami melon types (Xizhoumi No. 25, Dumilv No. 25), suggesting that Xizhoumi No. 25 and Dumilv No. 25 may carry certain muskmelon genetic backgrounds;

Group VI: Includes 2 Hami melon types and 1 honeydew type;

Group VII: Includes 3 cantaloupe types (Boge, Maliao, Xinniumeilong No. 3).

The clustering result is consistent with the PCA, further confirming that there is frequent germplasm exchange between the Hami melon and honeydew types, leading to their blurred genetic boundaries.

### 2.5. Construction of DNA Fingerprint Profiles

**Figure 7:**
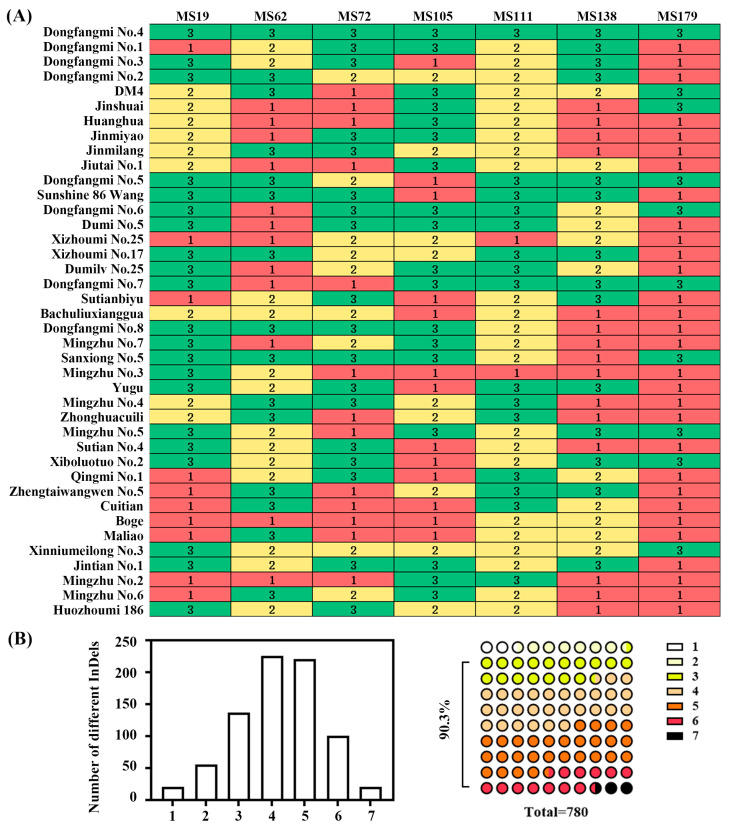
Construction and discriminative power analysis of DNA fingerprint profiles. (**A**) DNA fingerprint profiles of 40 melon varieties generated using seven core InDel markers (MS19, MS62, MS72, MS105, MS111, MS138, and MS179). (**B**) Statistical distribution of the number of different InDels for each of the seven markers in the order listed in (**A**), followed by the distribution of the difference in marker numbers in the 780 variety pairs. Note: In (**A**), 1 (green color) represents allele 1; 2 (yellow color) represents allele 2; 3 (red color) represents the simultaneous presence of both allele 1 and allele 2. In (**B**), the color in the right-hand 780 small circles corresponds to the number of shared InDel markers for each pair of varieties as listed and displayed with increasing intensity in the legend marked with small rectangles numbered 1 to 7 on the far right.

To distinguish and identify 40 melon varieties, seven core InDel markers (MS19, MS62, MS72, MS105, MS111, MS138, and MS179) were selected to construct DNA fingerprinting codes (refer to Table S4 for the genotype combinations of each marker). Utilizing these seven marker combinations, we successfully achieved complete differentiation among the 40 cultivated varieties (Figure 7A). Pairwise comparison analysis indicated that 90.3% of the possible variety pairs differed at three or more of the seven core InDel loci (Figure 7B). This finding demonstrates that this set of core InDel marker combinations possesses significant utility and discriminative power for the genetic identification of melon varieties.

## 3. Discussion

Genetic diversity is a crucial resource for species evolution and plant breeding [4,18]. Melon varieties are primarily distinguished by the morphology of mature fruits; however, identification at the seedling or seed stage presents challenges, as phenotypes are susceptible to environmental influences, leading to potential misjudgments [19]. Consequently, the development of rapid and accurate genotypic markers at the DNA level is essential for variety identification, seed purity testing, and breeding. In comparison to commonly used SSR and SNP markers, InDel markers can be analyzed using conventional PCR and agarose gel electrophoresis. These markers offer advantages such as lower costs, operational simplicity, stable results, and ease of interpretation [20,21], which hold significant application value for research and breeding efforts.

### 3.1. Distribution Characteristics of InDels and Their Value in Marker Development

This study identified 557,878 genome-wide InDel variations between the bi-parents of the melon variety ‘Dongfangmi No.4’, resulting in an average InDel density of 1486.25 InDels/Mb. The distribution of these InDels was generally uniform (Table S2). When compared with previous studies, our findings on InDel density and uniformity align with and extend existing knowledge. Oren et al. [22] conducted a pan-genome analysis and observed relatively uniform coverage of short InDels across melon chromosomes, corroborating our observation of a generally even distribution. However, the absolute density we report (1486.25 InDels/Mb) is specific to the parental lines of ‘Dongfangmi No.4’. Although the initial markers were developed from ‘Dongfangmi No.4’ and its bi-parental lines, they were designed to target conserved and polymorphic regions which may be transferable across thick-skinned melon varieties that are often challenging to differentiate morphologically.

In the context of melon breeding research, the development of InDel markers serves as an effective tool for marker-assisted selection of significant agronomic traits. Seven InDel markers (GX1-GX9) associated with fruit shape, derived from resequencing data, demonstrated an accuracy rate of 84.16–91.66% across 120 inbred lines, while 27 markers related to peel color (PS1-PS27) were validated within a genomic region of approximately 3.0 Mb. These markers have established a foundation for the genetic enhancement of fruit appearance traits [23]. Particularly noteworthy is the identification of the InDel marker chr06_indel_5 047 127, which is closely linked to resistance against powdery mildew, demonstrating direct practical value in breeding disease-resistant varieties [16]. The development of these molecular markers not only elucidate the genetic basis of important traits in melon but also provides technical support for molecular design breeding aimed at the coordinated improvement of multiple traits. Among the 234 InDel primer pairs initially designed, 39 polymorphic markers were successfully developed after stringent screening. It is worth noting that chromosomes 4 and 7, despite exhibiting higher InDel densities, yielded relatively fewer validated markers (Figure 1). This discrepancy may be attributed to local genomic features—such as repetitive sequences or complex structural variations in these regions—which often impede robust primer design and stable PCR amplification. Despite this, the marker MS108 (chr06_12951796) demonstrated precise identification of the hybrid variety ‘Dongfangmi No.4’ within the test panel (Figure 3), suggesting that this locus resides in a region of significant genetic differentiation and could serve as a molecular tool for variety protection within this specific set of materials. Future efforts will focus on further developing and optimizing markers in chromosomal regions with current low coverage, improving primer design strategies to overcome local genomic constraints, and validating marker performance across a broader range of melon germplasm to enhance the general applicability and robustness of this fingerprinting system.

### 3.2. The History of Breeding Exchanges Reflected by Population Structure

Geographical distance and selective pressure are two key factors influencing population genetic differentiation [24,25]. However, the 40 melon resources analyzed in this study, despite originating from nine different provinces in China, did not exhibit the anticipated geographical clustering pattern (Figure 5B). Firstly, the extensive introduction of germplasm by breeders and producers may have resulted in frequent exchanges and mixing of germplasm from diverse geographical origins, thereby diluting the original regional genetic differences. This occurrence has been reported in multiple crops; for instance, studies have indicated that the widespread dissemination of Kazakhstan melon germplasm in the eastern regions of the Silk Road has contributed to the mixing of genetic backgrounds [26]. Additionally, modern breeding practices that involve directional selection for specific traits may further accelerate the homogenization of genetic backgrounds. As an important economic crop, the continuous selection for desirable traits (such as disease resistance and fruit quality) in melon breeding may lead to the convergent evolution of germplasms from various geographical origins [3].

This study employs both principal component analysis and neighbor-joining clustering to reveal significant genetic differentiation between cantaloupe-type melons and the other two types (Hami melon and honeydew). Notably, Hami melon and honeydew varieties show mixed distribution across multiple subgroups; for instance, subgroup II contains five Hami melons and five honeydew melons (Figure 5A and Figure 6), suggesting extensive gene introgression between these two melon types during long-term breeding. Shigita et al. [27] investigated the genetic variation and population structure of 755 melon genetic resources from the NARO gene bank in Japan, finding that some Hami melon and honeydew varieties share a high proportion of SNP loci, indicating overlapping genetic backgrounds. Similarly, structural variation (SV) analysis revealed that 3317 highly differentiated SVs in cultivated melons were significantly different from those in wild species, although cantaloupes and honeydews may share some domestication-related SVs [28]. However, our study, focused on Chinese thick-skinned varieties, further highlights the significant differentiation between cantaloupe-type melons and other types (Hami and honeydew), a finding that may be more pronounced in this specific market segment compared to global germplasm surveys. Particularly noteworthy is that two honeydew materials (Xizhoumi No. 25 and Dumilv No. 25) in subgroup V clustered with cantaloupe (Figure 6), suggesting they might carry introgressed segments from cantaloupe or be influenced by convergent selection, providing genetic clues for the cross-type transfer of quality traits in melons.

### 3.3. Prospects for the Identification Application of Core Marker Combinations

This study successfully achieved the complete differentiation of 40 thick-skinned varieties, utilizing a DNA fingerprinting profile constructed from seven InDel markers. Notably, 90.3% of the variety pairs exhibited three or more differential markers (Figure 7). This marker system demonstrates high discriminative power, as only 2.56% of the variety pairs showed a single differential locus. Additionally, it offers operational convenience through PCR amplification, facilitating seamless integration into the DUS testing system. The efficiency of our core marker set in distinguishing thick-skinned varieties compares favorably with previous marker systems. For example, while SSR markers have been widely used, they often require more loci or capillary electrophoresis for similar resolution [8]. Our use of a small set (7) of InDel markers for complete discrimination highlights the advantage of targeting highly polymorphic sites preselected from parental sequencing data. Our core set complements these by providing a cost-effective, gel-based tool specifically optimized for identity and purity testing within a defined commercial group. While the current study specifically focused on thick-skinned varieties and the markers were developed from a particular hybrid combination, the high discrimination success rate suggests that these markers are particularly valuable for distinguishing closely related commercial varieties with morphological similarities.

While our core marker set demonstrates high discriminative power, we acknowledge that genotyping error is a potential concern, particularly when distinguishing accessions based on very few differential loci. The stringent laboratory protocols and replicate analyses employed here were designed to minimize such errors. For definitive identification, especially in legal or seed certification contexts, we recommend that a genotype match be based on a consensus across multiple loci. Future validation with larger and more diverse panels will further establish the error rate and robustness of this fingerprinting system.

## 4. Materials and Methods

### 4.1. Plant Materials

This study utilized a total of 40 melon varieties, which included 22 Hami melon types, 12 honeydew types, and 6 cantaloupe types. These melons were derived from various provinces across China and cultivated under diverse environmental conditions, with fruits harvested from March to August 2024. An analysis of the phenotypic characteristics of the different melon varieties was conducted, focusing on four traits: peel color, flesh color, fruit shape, and the presence of reticulation on the fruit surface, as detailed in Table 3.

**Table 3:** Information on 40 melon varieties and fruit phenotypes.

Number	Cultivar	Origin	Type	Peel Color	Pulp Color	Fruit Shape	Surface
1	Dongfangmi No.4	Shanghai	Hami melon	yellow	orange	oval	net
2	Dongfangmi No.1	Shanghai	Hami melon	white	orange	oval	smooth
3	Dongfangmi No.3	Shanghai	Hami melon	white	orange	oval	smooth
4	Dongfangmi No.2	Shanghai	Hami melon	yellow	orange	oval	net
5	DM4	Shanghai	Hami melon	yellow	orange	oval	net
6	Jinshuai	Xinjiang	Hami melon	yellow	orange	oval	net
7	Huanghua	Shandong	Hami melon	yellow	orange	oval	net
8	Jinmiyao	Xinjiang	Hami melon	yellow	orange	oval	net
9	Jinmilang	Hebei	Hami melon	yellow	orange	oval	net
10	Jiutai No.1	Fujian	Hami melon	yellow	orange	oval	net
11	Dongfangmi No.5	Shanghai	Hami melon	chartreuse	orange	oval	net
12	Sunshine 86 Wang	Xinjiang	Hami melon	chartreuse	orange	oval	net
13	Dongfangmi No.6	Shanghai	Hami melon	green	orange	oval	net
14	Dumi No.5	Shandong	Hami melon	green	orange	oval	net
15	Xizhoumi No.25	Xinjiang	Hami melon	Celadon green	orange	oval	net
16	Xizhoumi No.17	Xinjiang	Hami melon	Celadon green	orange	oval	net
17	Dumilv No.25	Shandong	Hami melon	Celadon green	green	oval	net
18	Dongfangmi No.7	Shanghai	Hami melon	Celadon green	green	oval	net
19	Sutianbiyu	Jiangsu	Hami melon	green	green	oval	net
20	Bachuliuxianggua	Xinjiang	Hami melon	green	green	oval	net
21	Dongfangmi No.8	Shanghai	Hami melon	black-green	white	fusiform	smooth
22	Mingzhu No.7	Shanghai	honeydew	yellow	green-white	circle	smooth
23	Sanxiong No.5	Zhejiang	honeydew	yellow	green-white	circle	smooth
24	Mingzhu No.3	Shanghai	honeydew	white	green	circle	smooth
25	Yugu	Fujian	honeydew	white	green	circle	smooth
26	Mingzhu No.4	Shanghai	honeydew	white	white	oval	smooth
27	Zhonghuacuili	Shandong	honeydew	white	white	oval	smooth
28	Mingzhu No.5	Shanghai	honeydew	white	white	circle	smooth
29	Sutian No.4	Jiangsu	honeydew	white	orange	oval	smooth
30	Xiboluotuo No.2	Shanghai	honeydew	white	white	circle	smooth
31	Qingmi No.1	Shanghai	cantaloupe	Celadon green	green	circle	net
32	Zhengtaiwangwen No.5	Shandong	cantaloupe	Celadon green	green	circle	net
33	Cuitian	Fujian	cantaloupe	Celadon green	green	circle	net
34	Boge	Shanghai	cantaloupe	Celadon green	green	circle	net
35	Maliao	Shanghai	cantaloupe	Celadon green	green	circle	net
36	Xinniumeilong No.3	Hainan	cantaloupe	Celadon green	green	circle	net
37	Jintian No.1	Anhui	honeydew	yellow	orange	oval	smooth
38	Mingzhu No.2	Shanghai	honeydew	white	white	circle	net
39	Mingzhu No.6	Shanghai	honeydew	yellow	white	circle	smooth
40	Huozhoumi 186	Hebei	Hami melon	yellow	orange	oval	net

### 4.2. Melon Material Planting and Sampling

The seeds of the 40 melon varieties were soaked in warm water at 50 °C for 12 h and then germinated in an incubator at 30 °C for a duration of 24 to 36 h. The germinated seeds were sown in 50-cell trays at the Shanghai Academy of Agricultural Sciences’ experimental station in Zhuanghang, Fengxian District, Shanghai (121°39′ E, 30°89′ N) in August 2024, with 5 to 6 seeds per variety. Standard water and fertilizer management practices were employed to ensure optimal germination and seedling emergence. The experimental station maintains an average temperature of 28 °C, an annual average sunshine duration of 1919.8 h, and an annual average rainfall of 1221.4 mm.

The young leaf tissues were sampled and used to exact the genomic DNA with CTAB method [29]. The concentration, OD260/OD280, and integrity of DNA were detected with Qubit 2.0 fluorometer (Invitrogen, Waltham, MA, USA), Nanodrop spectrophotometer (Thermo Fisher Scientific, Waltham, MA, USA), and 0.8% agarose gel electrophoresis, respectively. The qualified sample was used to subsequently re-sequence.

### 4.3. Genome-Wide Resequencing and Variants Calling

A total of 3 samples (‘Dongfangmi No. 4’ and its bi-parental lines) were utilized for resequencing on the Illumina platform, employing paired-end 150 bp reads with an insert size of 350 bp at Shanghai Ling’en Biotechnology Co., Ltd. (Shanghai, China). The quality control of the raw reads was performed using fastp (v0.23.2) with default parameters. Cleaned reads were aligned to the melon reference genome DHL92 (http://cucurbitgenomics.org/organism/18, accessed on 1 May 2024) using BWA-MEM (v0.7.17) with the ‘mem-M’ parameter. PCR duplicates were marked from alignment results using MarkDuplicates from Picard (v2.27.5). Insertions and deletions (InDels) were called with GATK (v4.2.6.1) with HaplotypeCaller model. Raw variants were filtered based on the following criteria:

(1) Homozygous loci in both parental lines;
(2) Genotypic differences between parental lines;
(3) Absence of missing genotypes in bi-parents;
(4) Heterozygous genotypes in offspring;
(5) Genotype quality (GQ) > q20.

### 4.4. InDel Primer Synthesis and Screening

A total of 234 InDel markers with mutation lengths ranging from 10 bp to 50 bp were randomly selected. Primers were designed using Primer Premier 5.0 software, focusing on the conserved regions flanking the differential sites. The lengths of the primers varied from 20 to 25 bp, while the PCR amplification products ranged from 200 to 320 bp, with annealing temperatures between 55 and 61 °C. These 234 pairs of InDel primers were synthesized by Sangon Biotech (Shanghai) Co., Ltd. (Shanghai, China) (Table S5). Subsequently, the primers were utilized to assess polymorphism in parental and F_1_ generation materials.

PCR was performed using the 2×Hiff@ PCR Master Mix (Yeasen Biotech, Shanghai, China) according to the following program: initial denaturation at 94 °C for 3 min, followed by 35 cycles of denaturation at 94 °C for 30 s, annealing at 60 °C for 30 s, and extension at 72 °C for 30 s, concluding with a final extension at 72 °C for 10 min. The PCR products were analyzed by electrophoresis on a 4% agarose gel [30], which was prepared with 2 g of agarose and 50 milliliters of TAE buffer, at a constant voltage of 100 volts for 80 min. Images were captured using a UV gel imaging system.

Primers were selected based on:

(1) clear, single-band amplification in both parents and F_1_;
(2) visible polymorphism (size difference) between parents;
(3) consistent Mendelian inheritance in the F_1_;
(4) absence of non-specific or stutter bands.

### 4.5. Data Analysis

The bands (0, 1 matrix) identified through agarose gel electrophoresis were statistically combined, and the PopGene 32 software was employed to calculate the number of alleles (Na), the effective number of alleles (Ne), the overall genetic diversity index, and Shannon’s index (I) to evaluate genetic diversity [31]. The polymorphism information content (PIC) and minor allele frequency (MAF) were calculated using PowerMarker version 3.25 [32]. Genetic distances were calculated based on the Dice Similarity Coefficient [33]. This was followed by hierarchical cluster analysis using the UPGMA algorithm as implemented in the NTSYSPC 2.10 software [34]] One-way ANOVA and other statistical comparisons were performed using SPSS 26 (Armonk, NY, USA: IBM Corp).

## 5. Conclusions

This study represents the first development of genome-wide InDel markers based on resequencing data of the parental lines of the thick-skinned melon variety ‘Dongfangmi No.4’. A total of 557,878 InDel loci were identified across the genome, from which 39 polymorphic InDel markers were screened and applied for genetic diversity analysis of 40 thick-skinned melon accessions. Notably, the marker MS108 (chr06_12951796) accurately distinguished ‘Dongfangmi No.4’ from other common cultivars within the tested panel, providing a specific molecular tool for variety authenticity and intellectual property protection. Furthermore, a core set of seven InDel markers demonstrated high discriminative power, enabling complete differentiation of all 40 varieties. The InDel marker system established herein offers a valuable technical foundation for variety identification, rights protection, and molecular breeding in thick-skinned melon.

## Figures and Tables

**Table 1:** Characteristics of the 39 polymorphic InDel markers used for genetic analysis.

Number	Position	Primer-F	Primer-R
MS6	chr01_7074391	TCCTTCTTTTTATCTTCTGCATGGC	CGGGTACAAGGCAACAGCTA
MS13	chr01_19733999	ACACATCAATATGATACATGGTCAAGT	TTCTCATTGGGGCCTTGTCC
MS14	chr01_19795754	GTTCTGTTTGTTGGGTCCACG	AGCTACCTCCATTGCGGAAG
MS19	chr01_33481852	TAGCGGGACAGAGTGTTTGG	GGGGCCCAAAGGATCAAAGA
MS21	chr01_34675645	TGGATTTACGATAAGAGGGCCT	TCCCCAAGTTGCAAGCTTGT
MS22	chr01_36018052	CGTGACATCCAAATTGATATTCGGT	CTCGACCTCAAGCCTCTGTC
MS30	chr02_11391369	CCAAGCTACCTAACACTTCCAAC	TGGAATTGTTGAGATGGGGGA
MS36	chr02_20541361	ACCAATGGCTCTATTTTATCTCCTCT	TTTTGCGGCTCTTTGTGCAA
MS39	chr02_26332867	TTGTCTTCGGATTCGGAAACCT	AGCATATCAGTGTCTATCGATGTCT
MS51	chr03_16330935	AGATTCGTGCTCAGGAAGTCA	ACAATGAAATCTCGTGCAAATTGC
MS58	chr03_29176371	AGGTTTTCTTACCAGGAAGCTACT	AGCACCCCATTGATTAAAAGCTG
MS59	chr03_31365283	CAAAGCATCGGATGGCTGAG	CGTACATTTTGTATCAACCAATGGC
MS62	chr04_1488958	CGACCAATAGTAGCTACCAACGA	TCGTAACTAAGCATTAATGAAAGCTGT
MS72	chr04_17954161	TGGTGCAAGGGATGAGAGTG	AATAGCGCTCCACGTGATGT
MS84	chr05_1320764	ACTTCACCGTCCTCCACAAG	ACCCAATTTTGTTCTCGAGTTTTCA
MS87	chr05_3963301	TGCAATGACCTTCAACGCTTC	TGGTGCAGCTTCGACTGATT
MS99	chr05_25658229	ACCTCATCAACTGTGAGGCC	AGGATGGAGCACTGATGAAGAA
MS105	chr06_6246570	GGTCCATTCAAGGCCTAGCA	TCCAGAAATCTCGTGTGAACAGA
MS108	chr06_12951796	CCCCTACCGTGCTCAAACTT	GTCCTTCGTTTCATTGGCGG
MS111	chr06_16084050	GGGATAGGCTCGGCTTGATT	CCGTGGTTTTAGTCGGACCA
MS117	chr06_24013568	AATCCTCCATTGGGCCAGTC	ACTGGACGTCCTAAAAAGCGA
MS129	chr07_1885541	CCAGCCGTGAAAGTAAAATAAAACG	GCACACATGCCATGGATAACA
MS136	chr07_14576627	CCCTTGACCAAAGTTCGTGG	AGAATTGAGTTGTGGGACCCA
MS138	chr07_18178680	TGATTGAGGGAGGAAGAGGGA	TCTGTCGTCCTGTGGTTGTG
MS139	chr07_18211629	AGGCGTTCAAAAGAAGGGAAC	TGTGGTCCTTTCACCCCTTG
MS153	chr08_12448261	TGGCGAAGATGAAGTTGGGT	TGGGGCGTTACATTCTTCGAT
MS154	chr08_12700987	TGTCCAACACATGCAAACGG	TCCCTGTGTTCGACCCTAGAT
MS155	chr08_14175099	AGAAGGTGTGGAGAAGCACC	TGTTCGCCGTCTCTTGTTCT
MS170	chr09_3616045	CAATTTCGCAGGGAGAGGGA	GCAGTGCCATGGCTTCAAAT
MS172	chr09_6721132	GCAACCAATAAGTTCCTGCCA	AGCATCAAACATCATTGACTAGGG
MS178	chr09_19253495	GTTGGCGACCAAACAACTCC	ATGTGTGTGTGTGTGAGCCT
MS179	chr09_21200612	GTTGGATGATGGAGGAGAAGCT	AGTGGGAAGGAAAGTTGCACT
MS185	chr10_1552627	TCAATAGTGCCACGTGGGG	ACGCAAAGTTGAAACCGACC
MS194	chr10_18050725	GAGTGGCAGTACTCAAGCTCA	TGGCAGTGAAGGGAAGAGAAG
MS196	chr10_21741047	TGGATGGAGAAGCCCTCTTTG	AGCTCACGACACTCAAGTTGT
MS205	chr11_6850271	TGAACTGTAGTCATAAGGTGTACTCAA	TCTTGTTGGACTTGAGCACTCA
MS217	chr11_29625033	AGCATCACTTGGTCTAGCTTCT	CCTGTGTATGGGGAAGTGCA
MS225	chr12_8076169	CAACTCCAACGTTTCGTCCG	GTCCTCGGCTCGGATCAATT
MS233	chr12_24555285	GGCCCTGTCTCTCTGTTACC	AAGGAAAAGGTTGGAAGCGC

The markers are listed by their assigned laboratory codes (MS number) for consistent reference in this study.

**Table 2:** Results of the genetic analysis of the 39 InDel markers.

Order	Na	Ne	He	Ho	I	PIC	MAF
MS6	2.000	1.406	0.289	0.300	0.464	0.454	0.175
MS13	2.000	1.406	0.289	0.300	0.464	0.454	0.175
MS14	2.000	1.663	0.399	0.300	0.588	0.564	0.275
MS19	2.000	1.999	0.500	0.525	0.693	0.611	0.488
MS21	2.000	1.941	0.485	0.475	0.678	0.621	0.413
MS22	2.000	1.995	0.499	0.500	0.692	0.624	0.475
MS30	2.000	1.663	0.399	0.400	0.588	0.558	0.275
MS36	2.000	1.956	0.489	0.650	0.682	0.505	0.425
MS39	2.000	1.941	0.485	0.575	0.678	0.564	0.413
MS51	2.000	1.311	0.237	0.175	0.400	0.366	0.138
MS58	2.000	1.051	0.049	0.050	0.117	0.095	0.025
MS59	2.000	1.600	0.375	0.300	0.562	0.540	0.250
MS62	2.000	1.999	0.500	0.375	0.693	0.664	0.488
MS72	2.000	1.980	0.495	0.450	0.688	0.641	0.450
MS84	2.000	1.280	0.219	0.200	0.377	0.359	0.125
MS87	2.000	1.835	0.455	0.300	0.647	0.620	0.350
MS99	2.000	1.438	0.305	0.275	0.483	0.466	0.188
MS105	2.000	1.980	0.495	0.450	0.688	0.641	0.450
MS108	2.000	1.025	0.025	0.025	0.067	0.048	0.013
MS111	2.000	1.568	0.362	0.375	0.548	0.526	0.238
MS117	2.000	1.859	0.462	0.475	0.655	0.599	0.363
MS129	2.000	1.632	0.387	0.275	0.576	0.549	0.263
MS136	2.000	1.923	0.480	0.450	0.673	0.626	0.400
MS138	2.000	1.980	0.495	0.350	0.688	0.661	0.450
MS139	2.000	1.882	0.469	0.100	0.662	0.304	0.125
MS153	2.000	1.250	0.200	0.075	0.352	0.266	0.113
MS154	2.000	1.220	0.180	0.150	0.325	0.296	0.100
MS155	2.000	1.753	0.430	0.275	0.621	0.591	0.313
MS170	2.000	1.438	0.305	0.325	0.483	0.471	0.188
MS172	2.000	1.250	0.200	0.225	0.352	0.349	0.113
MS178	2.000	1.406	0.289	0.200	0.464	0.429	0.125
MS179	2.000	1.280	0.219	0.250	0.377	0.375	0.175
MS185	2.000	1.406	0.289	0.150	0.464	0.405	0.175
MS194	2.000	1.406	0.289	0.250	0.464	0.445	0.125
MS196	2.000	1.904	0.475	0.425	0.668	0.628	0.388
MS205	2.000	1.471	0.320	0.250	0.500	0.476	0.200
MS217	2.000	1.989	0.497	0.525	0.690	0.609	0.463
MS225	2.000	1.374	0.272	0.275	0.444	0.139	0.038
MS233	2.000	1.250	0.200	0.175	0.352	0.595	0.475
Mean	2.000	1.608	0.354	0.313	0.528	0.480	0.267

Na = Number of Alleles; Ne = Effective Number of Alleles; He = Expected Heterozygosity; Ho = Average Observational Heterozygosity; I = Shannon’s index; PIC = Polymorphism Information Content; MAF = Minor Allele Frequency.

## Data Availability

The datasets presented in this study can be found in online repositories. These datasets have been deposited in the Genome Variation Map in National Genomics Data Center, Beijing Institute of Genomics, Chinese Academy of Sciences and China National Center for Bioinformation, under accession number GVM001145 (https://ngdc.cncb.ac.cn/gvm/getProjectDetail?project=GVM001145, accessed on 1 September 2025).

## Supplementary Materials

The following supporting information can be downloaded at: https://www.mdpi.com/article/10.3390/plants14243782/s1, Table S1. The length distribution of 557,878 InDel variants. Table S2. Length (bp), Number of InDels, and Genome-wide Density of Indels (/Mb) for each of the 12 chromosomes. Table S3. Amplification products of MS108 primer. Table S4. The genotype combinations of nine core InDel markers. Table S5. Primers for detecting 234 InDel markers.
